# Epidermal Growth Factor Receptor Tyrosine Kinase Inhibitors and Lung Cancer: History, Epidemiology, and Market Outlook

**DOI:** 10.7759/cureus.13470

**Published:** 2021-02-21

**Authors:** Brittany Miles, James D Mackey

**Affiliations:** 1 Medical Education, University of Texas Medical Branch, Galveston, USA; 2 Medical Oncology, Baylor University Medical Center, Dallas, USA

**Keywords:** tyrosine kinase receptor inhibitors, epidermal growth factor receptor gene mutation, metastatic non-small cell lung cancer, t790m

## Abstract

Lung cancer is a leading cause of death for both men and women. The treatment of lung cancer has been stifled with pessimism for many years. However, molecularly targeted therapies directed at pathologic epidermal growth factor receptor tyrosine kinases have come to market and, with them, a new tone to an old diagnosis. Treatment of lung cancer is a complex science that requires not only anatomical knowledge of the patient but also an understanding of the patient's overall physiologic condition. When patients are treated appropriately, this drug can transform the natural progression of their disease and improve survival. Interestingly, the clinical solidarity of these first-generation tyrosine kinase inhibitors (TKIs) has become the prelude to a second wave of advances in molecular targeting that we can expect to further improve how we classify and treat lung cancers. Cancers that become resistant to epidermal growth factor receptor (EGFR)-specific TKIs through a secondary mutation are likely to be dependent on the activated kinase for their growth and survival. Therefore, discovering a secondary means of inhibiting EGFR T790M may be therapeutically necessary. This has prompted the preclinical and clinical development of second and third-generation kinase inhibitors. Tumor subtypes are also now being identified, potentially allowing patients to be treated with drugs that most benefit their tumor subtype. We used the TriNetX research platform to analyze the rate of patients being prescribed first, second, and third-generation EGFR TKIs and propose a rationale for the trends seen over time.

## Introduction and background

Lung cancer is a leading cause of death for both men and women. The treatment of lung cancer has been stifled with pessimism for many years. However, molecularly targeted therapies directed at pathologic epidermal growth factor receptor (EGFR) tyrosine kinases have come to market, and with them came a new tone to an old diagnosis. The treatment of lung cancer is a complex science that requires not only anatomical knowledge of the patient but also an understanding of the patient's overall physiologic condition. When patients are treated appropriately, these drugs can transform the natural progression of their disease and improve survival.

EGFR tyrosine kinase mutations are a well-established driver of proliferation in non-small cell lung cancer (NSCLC) and are present in approximately 15% of patients with lung adenocarcinoma in the United States, albeit with greater frequency in women and nonsmokers. Populations from Asian countries have an incidence of EGFR mutation that is substantially higher; in one study, 51.4%. Asian nonsmokers were more likely to be found with an EGFR mutation than smokers, but the incidence in regular smokers was still 37% [1]. The story for Caucasian patients is very different, where the incidence is only 7%-17% and is most commonly associated with never-smokers that have adenocarcinoma [2-3].

The first patient treated with an EGFR tyrosine kinase inhibitor (TKI) received ZD1839, later named gefitinib, in April 1998. Of the initial 16 patients treated, four eventually showed objective partial responses on doses of 300-700 mg daily [4]. It became apparent in phase II studies that patients were more likely to respond if they were female, never-smokers, had adenocarcinoma, or were of Asian descent but the reason behind these correlations was not known. Gefitinib initially received Food and Drug Administration (FDA) approval for lung cancer in 2003 with the caveat that patients must have progressed on both platinum-based and docetaxel chemotherapies [5]. The approval was based on a modest 13.6% response rate in phase II clinical trials. In 2004, two papers reported that the presence of an EGFR mutation appeared to be linked to dramatic tumor response with gefitinib, but the magnitude of that discovery was not realized, and the standard-of-care was for patients to be treated without biomarker selection [6-7]. 2004 also saw the newcomer, first-generation TKI erlotinib, receive FDA approval for patients with locally advanced or metastatic NSCLC after treatment with one prior chemotherapy regimen, but the overall response rate of erlotinib in that setting was also very modest at 8.9%. Gefitinib was withdrawn from the US market in 2005 due to perceived ineffectiveness, as patients at that time were still being treated in an unselected fashion without prior biomarker testing for EGFR mutation status. The landmark phase III I-PASS study was presented at the European Society for Medical Oncology in 2008 and published in 2009 and convincingly demonstrated that the presence of an activating EGFR mutation was the best biomarker for predicting response to TKI therapy. Patients receiving gefitinib compared to doublet chemotherapy had a hazard ratio of 0.48 with a P-value of <0.0001. This ultimately led to the use of erlotinib in the United States for first-line therapy for patients with EGFR mutations, and FDA approval for this setting occurred in 2013.

As researchers were starting to better understand the role of EGFR mutation as a driving force for some lung cancers, it was also being realized that an acquired resistance mutation, T790M, was responsible for many cases of treatment failure on first-generation agents [8]. The need to overcome the T790M resistance mutation led to the development of second-generation EGFR TKIs, the first of which was afatinib, approved for first-line use by the FDA in 2013 [9].

Finally, in 2018 osimertinib (third generation) and dacomitinib (second generation) would receive FDA approval for first-line use in EGFR-driven NSCLC as well [10]. Osimertinib has shown great activity against EGFR driver mutations, at the same time sparing wild-type EGFR and overcoming the resistance mutation T790M [11]. Unfortunately, newly acquired resistance mutations are being discovered in patients that progress on osimertinib so the cat and mouse game continues.

As an aside, gefitinib belatedly returned to the US market in 2015, this time with biomarker testing as a prerequisite [12]. The reintroduction of gefitinib to a market that had already moved on from first-generation drugs was primarily intended to provide an additional treatment option for patients that may not be candidates for standard chemotherapy agents.

Third-generation osimertinib has been shown in the first-line setting to have better progression-free and overall survival as compared to first-generation TKIs [13]. It irreversibly binds the epidermal growth factor receptor and inhibits both sensitizing and T790M resistance mutations. For these reasons, it is the preferred first-line agent recommended by the National Comprehensive Cancer Network, with first and second-generation TKIs remaining in the category of “Other Recommended.” We set out to analyze the prescribing behavior of osimertinib compared to the first and second-generation TKIs using a global research database, TriNetX.

## Review

We used TriNetX, a global federated health research network providing access to electronic medical records (diagnoses, procedures, medications, laboratory values, genomic information) from approximately 212 million patients in 92 large healthcare organizations. The TriNetX platform only uses aggregated counts and statistical summaries of de-identified information. No protected health information (PHI) or personal data is made available to the users of the platform. We created three cohorts for analysis, one for each generation of TKI used for patients with a lung cancer diagnosis. The cohort for third-generation TKI included only osimertinib, as it is the only third-generation drug available at the time of this study. Data were available for the 35 months prior to July 2020, and graphs of prescribing behavior were generated using the TriNetX system.

**Figure 1:**
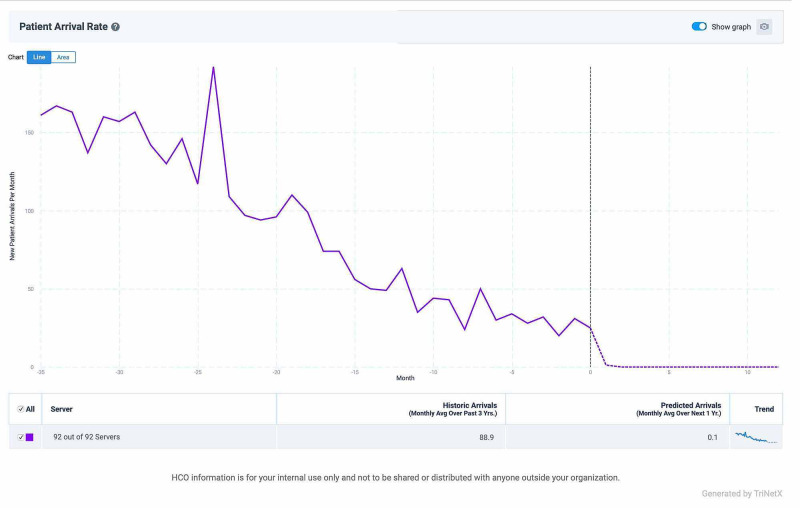
Declining incidence of new patients placed on first-generation TKIs TKI: tyrosine kinase inhibitor

We found that prescriptions for first-generation TKIs in NSCLC have decreased dramatically over the previous 35 months, and the predictive model of TriNetX indicates that very few prescriptions are likely to occur in the future (Figure 1).

**Figure 2:**
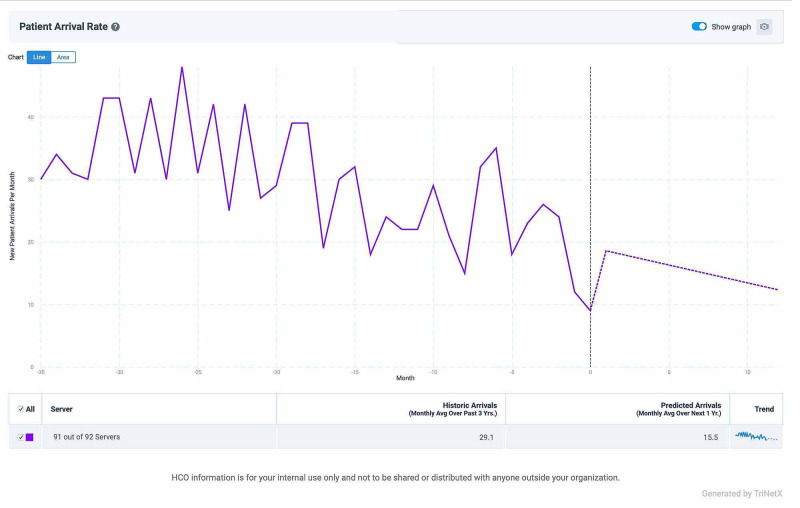
Declining incidence of new patients placed on second-generation TKIs TKI: tyrosine kinase inhibitor

Second-generation TKIs showed a relatively stable rate of utilization, but they started to decline steadily starting mid-2018 (Figure 2). The historic monthly number of new patients prescribed a second-generation TKI was 29, but modeling predicts that the next 12 months will see a 47% decrease.

**Figure 3:**
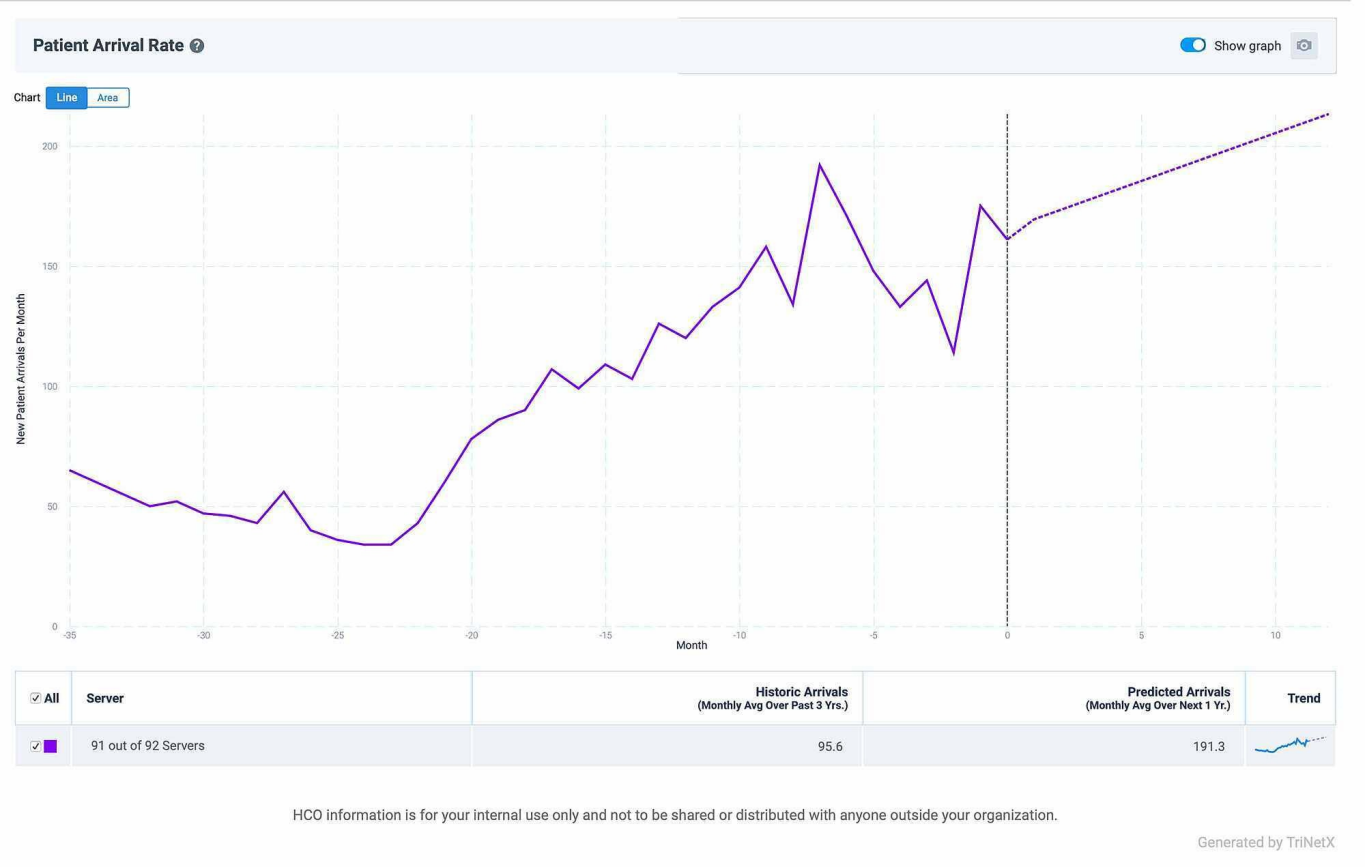
Increasing incidence of new patients placed on third-generation TKIs TKI: tyrosine kinase inhibitor

April 18, 2018, was the initial date of FDA approval for osimertinib in the United States, and as the first and second-generation TKIs were decreasing in use over this time period, third-generation osimertinib started to show a significant increase. This is not surprising, since the preliminary results of the FLAURA study were presented in the January 11, 2018 edition of the New England Journal of Medicine, and osimertinib showed an impressive improvement in median progression-free survival compared to first-generation TKIs (18.9 vs 10.2 months) [14]. This improvement in PFS also came with a decrease in serious adverse events. The final results of the FLAURA trial were released at the European Society for Medical Oncology (ESMO) 2019, and overall survival was shown to be superior at 35.8 months vs. 27.0 months [15]. The TriNetX predictive model estimates that the use of osimertinib should double over the next 12 months (Figure 3).

 The first-generation EGFR tyrosine kinase inhibitors represented a breakthrough for a subset of patients with non-small cell lung cancer. Gefitinib and erlotinib are effective therapies for non-small cell lung cancer patients with tumor mutations in EGFR that do not confer resistance. However, all patients develop resistance to these agents eventually [16-19]. Two primary mechanisms of acquired resistance have been identified. The first is a secondary EGFR mutation, T790M, which renders gefitinib and erlotinib ineffective inhibitors of EGFR kinase activity. This pathway is estimated to be seen in about 50% of tumors that are resistant to gefitinib and erlotinib [20-21]. The EGFR T790M mutation occurs in a position analogous to known resistance mutations to imatinib in other kinases (T315I in ABL, T674I in PDGFRA, and T670I in KIT) [15-17]. The conserved threonine residue among these different kinases, located near the kinase active site, is often referred to as the gatekeeper mutation [22].

## Conclusions

Second-generation drugs built on the progress of the first generation, with irreversible binding and improved efficacy in addition to an improved side-effect profile and the ability to overcome T790M resistance mutations. Third-generation EGFR TKIs represent even more progress. With mutation-specific binding and improved sparing of wild-type EGFR, the third-generation drugs offer the best efficacy and most favorable side-effect profile yet. It is therefore logical and expected that the use of first and second-generation EGFR TKIs would decrease over time as their continued use becomes more difficult to justify.
